# Interactions Screenings Unearth Potential New Divisome Components in the *Chlamydia*-Related Bacterium, *Waddlia chondrophila*

**DOI:** 10.3390/microorganisms7120617

**Published:** 2019-11-26

**Authors:** Firuza Bayramova, Nicolas Jacquier, Gilbert Greub

**Affiliations:** Center for Research on Intracellular Bacteria, Institute of Microbiology, University Hospital Center and University of Lausanne, Bugnon 48, CH-1011 Lausanne, Switzerland; firuza.bayramova@chuv.ch (F.B.); nicolas.jacquier@chuv.ch (N.J.)

**Keywords:** *Waddlia chondrophila*, *Chlamydia*-related bacteria, *Chlamydiales*, cell division, MreB, RodZ, peptidoglycan, cell wall

## Abstract

*Chlamydiales* order members are obligate intracellular bacteria, dividing by binary fission. However, *Chlamydiales* lack the otherwise conserved homologue of the bacterial division organizer FtsZ and certain division protein homologues. FtsZ might be functionally replaced in *Chlamydiales* by the actin homologue MreB. RodZ, the membrane anchor of MreB, localizes early at the division septum. In order to better characterize the organization of the chlamydial divisome, we performed co-immunoprecipitations and yeast-two hybrid assays to study the interactome of RodZ, using *Waddlia chondrophila*, a potentially pathogenic *Chlamydia*-related bacterium, as a model organism. Three potential interactors were further investigated: SecA, FtsH, and SufD. The gene and protein expression profiles of these three genes were measured and are comparable with recently described division proteins. Moreover, SecA, FtsH, and SufD all showed a peripheral localization, consistent with putative inner membrane localization and interaction with RodZ. Notably, heterologous overexpression of the abovementioned proteins could not complement *E. coli* mutants, indicating that these proteins might play different functions in these two bacteria or that important regulators are not conserved. Altogether, this study brings new insights to the composition of the chlamydial divisome and points to links between protein secretion, degradation, iron homeostasis, and chlamydial division.

## 1. Introduction

*Chlamydiales* are Gram-negative, obligate intracellular bacteria sharing a unique biphasic developmental cycle. Chlamydial division has been a mystery for a long time due to its minimal division machinery, which lacks several division proteins that are essential in other bacteria. This reduced division machinery is conserved among members of the *Chlamydiales* order, both in the well-described *Chlamydiaceae* family and as in *Chlamydia*-related bacteria [1]. The *Chlamydiaceae* family includes several well-known human pathogens, such as *Chlamydia trachomatis*, *Chlamydia pneumoniae, and Chlamydia psittaci. C. trachomatis* is a strict human pathogen which is the leading bacterial cause of sexually transmitted infections [2] and the causative agent of trachoma, an eye infection that can lead to blindness [3]. *C. pneumoniae* and *C. psittaci* infections can lead to respiratory tract infections in humans, such as pneumonia [4], bronchitis [5], and psittacosis, respectively [6]. The *Chlamydia*-related bacterium *Waddlia chondrophila*, from the *Waddliaceae* family, is suspected to play a role in abortion in ruminants [7,8,9] and miscarriage in humans [2,10,11]. Furthermore, the presence of *W. chondrophila* is associated with lower respiratory tract infections. Indeed, *W. chondrophila* DNA was detected in nasopharyngeal samples from children with bronchitis [12] and in respiratory samples from patients with pneumonia [13]. 

The chlamydial biphasic developmental cycle is conserved among the *Chlamydiales* order. It is characterized by two different bacterial morphologies: infectious nondividing elementary bodies (EBs) and noninfectious dividing reticulate bodies (RBs) [14,15]. EBs enter the host cell by phagocytosis or endocytosis, thus being first engulfed in an endosome vesicle, which, after several modifications induced by the bacterium itself, becomes an inclusion. Typically, *W. chondrophila* recruits mitochondria around its inclusion within three hours post-infection and escapes the endocytic pathway by maturing the inclusion in a vacuole expressing endoplasmic reticulum proteins, such as calnexin [16]. After several cycles of replication, RBs redifferentiate into EBs and leave the host cell through exocytosis or cell lysis [15]. Under certain conditions, *Chlamydiales* can enter a persistent nondividing, noninfectious stage called aberrant bodies (ABs). Diverse stimuli can induce the formation of aberrant bodies: addition of ß-lactam antibiotics such as penicillin, clavulanic acid [17], phosphomycin [18], iron or nutrient starvation [19], IFN-gamma treatment [19], and co-infection of the host with herpes or other viruses [20].

In order to better describe the chlamydial division mechanism, *Chlamydia*-related bacterium *W. chondrophila* was used as a model organism. Several reasons make *W. chondrophila* a convenient model for this study. First of all, *W. chondrophila* can infect and proliferate in a wide range of host cells, such as Vero cells, amoebae, human macrophages, pneumocytes, endometrial cells, insect cells, and fish cell lines [21,22,23]. Furthermore, *W. chondrophila* was shown to exhibit a large genome, which makes it interesting for the development of genetic tools and studying *Chlamydiales* evolution [24]. Next, *W. chondrophila* cells are larger in size, making them eligible for microscopic observations, especially for tracking protein localization during chlamydial division. Last but not least, unlike *Chlamydiaceae, W. chondrophila* has been shown to be sensitive to phosphomycin, which targets the very first step of peptidoglycan (PG) biosynthesis [25].

PG is an essential component of the bacterial cell wall and is composed of a chain of alternating molecules called N-acetylglucosamine (GlcNAc) and N-acetylmuramic acid (MurNAc) residues that are cross-linked by short peptides made of L- and D-amino acids. PG maintains the bacterial shape, protects bacteria from environmental stress, provides them with a structural strength, and is involved in bacterial division [26]. Members of the *Chlamydiales* order were initially thought to lack PG, whereas recent studies detected PG in several members of *Chlamydiaceae* and *Chlamydia*-related bacteria. Intriguingly, the PG-like material resides mainly at the chlamydial division septum in *Chlamydiaceae* [27,28,29].

*Chlamydiales* possess a minimal division machinery, as they lack the main organizer of bacterial division FtsZ and several additional division septum proteins. FtsZ is a tubulin homologue and the main organizer of the cytokinetic platform in the majority of prokaryotic cells. The main function of FtsZ is to assemble a stable but dynamic cytokinetic ring (Z ring) at the future site of division and to recruit other components of the division apparatus (the “divisome”) [30]. Thus, the main function of the divisome components in *Chlamydiales* is the modification and synthesis of PG [26]. Apparently, in the absence of an FtsZ homologue, *Chlamydiales* still divide by binary fission [24,31]. Presumably, in *Chlamydiales,* the tubulin FtsZ has been replaced by the actin homologue MreB, which borders the cytoplasmic membrane and is involved in PG synthesis during elongation of rod-shaped bacteria [32,33,34].

Recent studies proposed that *C. trachomatis* relies on rod-shaped determining proteins Pbp2 and MreB for cell division [35]. Indeed, application of MreB inhibitors could arrest *C. trachomatis* division and induce formation of aberrant bodies [35]. The actin homologue MreB was also shown to define the predicted septal plane during chlamydial division in *C. trachomatis* [36]. Moreover, recent studies have also demonstrated that *W. chondrophila* relies on the actin homologue MreB and its regulator, RodZ, for division [18]. Interestingly, MreB was detected at the division septum during middle and late division stages, whereas its regulator, RodZ, was shown to be an early recruit [18]. Another septal protein playing an important role in *W. chondrophila* cell division and primarily in PG remodeling is NlpD. NlpD was shown to be localized at the *W. chondrophila* division septum as an intermediate recruit [18,37]. Moreover, *C. pneumoniae* NlpD was shown to have a carboxypeptidase activity in vitro and is important for the PG remodeling [38]. Another protein called SpoIID was recently identified as a member of chlamydial divisome that participates in the PG remodeling during division [39]. Remarkably, *C. trachomatis* possesses only three annotated cell division genes, *ftsI, ftsK,* and *ftsW,* whereas two additional cell division genes, *ftsL* and *ftsQ,* were detected in the *W. chondrophila* genome [26].

This lack of conserved divisome components might be explained by (i) the presence of a minimal division machinery composed of only a few proteins or (ii) the functional replacement of division proteins by unrelated proteins. In order to investigate these hypotheses, we aimed to further characterize the composition of the chlamydial division machinery, in order to describe potential new chlamydial divisome members. We hypothesized that RodZ, as a membrane anchor of MreB and an early recruit of chlamydial division might be a potential organizer of chlamydial division and might interact with other divisome components. A split-ubiquitin Yeast-Two-Hybrid screen was thus performed, with RodZ as a bait [40]. Among the interactor candidates, SecA, SufD, and FtsH proteins were selected for further studies based on their potential links with division in other bacteria. We show here that their expression pattern and localization are consistent with a putative role in division. In a further step, complementation studies in *E. coli* indicated that these proteins might have distinct functions in *W. chondrophila* compared to *E. coli*.

## 2. Materials and Methods

### 2.1. Antibodies, Drugs, and Reagents

Polyclonal mouse antibodies against *W. chondrophila* were produced by our group, as previously described [16]. Secondary antibodies, Goat anti-mouse green Alexa 488, and Goat anti-rabbit red Alexa 594 were purchased from Thermo Fischer Scientific (Waltham, MA, USA). DAPI was obtained from Molecular Probes (Grand Island, NY). The antibiotics penicillin and phosphomycin, and 2, 2-Bipyridyl were purchased from Sigma-Aldrich (St Louis, MO, USA).

### 2.2. Split-Ubiquitin Yeast-Two-Hybrid Screening

Yeast-two-hybrid (Y2H) was performed, following the DUALhunter kit protocol (Dualsystems Biotech, Schlieren, Switzerland). Briefly, RodZ encoding gene was cloned in a pDHB1 vector in fusion with the Ost4 membrane anchor and the C-terminal part of ubiquitin (Bait plasmid). Following instructions provided by the kit manufacturer, the bait plasmid was introduced in a *Saccharomyces cerevisiae* strain (NMY51), and control assays were performed to verify the correct expression of the bait and absence of autoactivation in the presence of an empty prey vector (pPR3-N) with an addition of 3-AT, to increase screening stringency. A genomic library of *Waddlia chondrophila* was then created in the prey vector pPR3-N by fragmentation of genomic DNA and cloning in pPR3-N, in the presence of linkers of different lengths, to cover all three possible reading frames (Proteinlinks, Pasadena, CA).

Plasmids were co-transformed in *Saccharomyces cerevisiae,* and positive interactors were selected by growth on selective medium. Prey inserts of positive clones were amplified by PCR and sequenced. Positive hits were confirmed by reintroduction of the corresponding prey plasmid, together with the bait in NMY51 and growth on selective plates.

### 2.3. Mammalian Cell Culture and Bacterial Infection 

Vero cells (ATCC CCL-81) were grown in 75 cm^2^ flasks with 20 mL DMEM containing 10% fetal calf serum, at 37 °C, in the presence of 5% CO_2_. Cells were then detached, counted, diluted to 2 × 10^5^ cells/mL, and grown overnight. The next day, cells were infected with a 2000 dilution of *W. chondrophila* (ATCC VR-1470T, grown in *Acanthamoeba castellanii* ATCC 30010). The cells were then centrifuged for 15 min at 1790× *g*, incubated 15 min at 37 °C, washed with PBS, and supplemented with fresh media.

### 2.4. Quantitative PCR 

Gene expression was quantified by quantitative reverse-transcription PCR (qRT-PCR). Infected Vero cells were grown in 24-well plates at 37 °C. Infected cells were scrapped at 24, 32, 48, and 72 hours post infection, and 500 µL of cell suspensions was mixed with 1 mL of RNA Protect (Qiagen, Venlo, Netherlands), vortexed for 5 min, and then incubated for 5 min, at room temperature. The samples were then centrifuged for 5 min at 10,000× *g*. The supernatant was removed and the pellet was kept at –80 °C. Remaining DNA was eliminated, using the Ambion DNA-free kit™ (Life technologies). The retrotranscription was performed, using the GoScriptTM Reverse Transcription System (Promega). The qRT-PCR was performed, using iTaq supermix with ROX (BioRad, Hercules, CA). *W. chondrophila* primers, WadF4 and WadR4 targeting the 16S rRNA [12], and primers specific for the genes of interest (Table S1). Cycling conditions were 3 min at 95 °C, followed by 45 cycles of 15 s at 95 °C, and 1 min at 60 °C on a StepOne Plus Realtime PCR System (Applied Biosystems, Carlsbad, CA).

### 2.5. Protein Extraction and Immunoblotting

At different time points after infection, infected cells were scrapped, and samples of 500 µL were taken after homogenization of the cells and cell supernatant. The samples were washed in PBS and centrifuged, and then the proteins were solubilized and denatured by resuspension in 100 µL of sample buffer (60 mM Tris, pH 6.8, 1% SDS, 1% mercaptoethanol, 10% glycerol, 0.02% bromophenol blue) and by incubation at 95 °C for 5 min. Next, 10 µL of each sample was loaded on a Precast Protein Gel (12% polyacrylamide, Bio-Rad). The migration was performed at 200 V, with 35 mA per gel, for 45 min, in migration buffer (30 g/L tris(hydroxymethyl)aminomethane, 144g/l glycine, 0.1% sodium dodecyl sulphate). Following migration, the proteins were transferred onto a nitrocellulose membrane (GE Healthcare) in transfer buffer (3 g/Ltris(hydroxymethyl)aminomethane, 14.4 g/L glycine, 40% methanol) by electroblotting at a constant voltage of 75 V and 200 mA for 1 hour. The membrane was blocked in a saturation buffer containing 5% nonfat dry milk, at room temperature, for 2 hours. Next, the primary antibodies were diluted in saturation buffer with 0.5% nonfat dry milk (1:200 dilution of mouse anti SecA, FtsH, and SufD antibodies) and incubated at room temperature for 2 hours. Afterward, the membrane was washed 3 times, for 5 min, in saturation buffer supplemented with 0.5% milk. Following the washes, the membrane was incubated for 2 hours with the secondary antibody, goat anti-mouse IgG (H+L)–HRP Conjugate (Bio-Rad). The membrane was then treated with the Amersham™ ECL™ Prime Western Blotting Detection Reagent (GE Healthcare, Chicago, IL). To record the chemiluminescence, ImageQuant LAS 4000 Mini Imager (GE Healthcare) was used. After detection, the images were treated with the ImageJ software (www.macbiophotonics.ca).

### 2.6. Immunofluorescence Labeling

Infected Vero cells on glass coverslips were fixed with ice-cold methanol for 5 min, at room temperature. After fixation, cells were washed three times with PBS and then blocked and permeabilized for at least 1 h with a blocking buffer (PBS, 0.1% saponin, 1% BSA). For double immunostaining, the samples were incubated in blocking solution for 1 hour, at room temperature, with 1:1000 dilution of primary rabbit anti *W. chondrophila* antibodies and 1:200 mouse antibody dilutions targeting the protein of interest. After three washes with PBS, coverslips were incubated for 1 hour in blocking solution containing 1:1000 dilutions of secondary antibodies, Goat anti-mouse green Alexa 488 and Goat anti-rabbit red Alexa 594 (Thermo Fischer, Waltham, MA, USA), and 150 ng/mL DAPI (Molecular Probes). Coverslips were washed three times with PBS, once with water, and were mounted onto glass slides, using Mowiol (Sigma-Aldrich).

### 2.7. Confocal and Fluorescence Microscopy

Protein localization in aberrant bodies was examined by confocal microscopy, using a Zeiss LSM 510 Meta microscope (Zeiss, Oberkochen, Germany). Images were treated with the ImageJ software.

### 2.8. E. coli Growth Measurement

*E. coli* EC100D was grown in Luria Bertani broth (LB). Genes of interest were amplified and inserted in a pSRK-Gm vector using standard method (digestion-ligation cloning and heat-shock transformation) [41]. Overnight cultures were diluted to an absorbance at 600 nm (OD_600_/mL) of 0.2. The diluted cultures were incubated for 1 hour, to exponential phase, and induced by 1 mM of IPTG (AppliChem), but no IPTG was added to the controls. Bacterial growth was recorded by measuring the optical density of bacteria every 2, 4, and 6 h, using a spectrophotometer.

### 2.9. E. coli Morphology Observations

Cell cultures were treated as for growth measurements. After 6 hours of incubation, in the presence or absence of an IPTG inducer, 100 µL of the culture was centrifuged for 3 min, at 16,000× *g*. The pellet was resuspended in 20 µL of the supernatant, and 5 µL of this was put onto a glass slide and covered with a coverslip. The observations were performed with a Zeiss Axioplan 2 Imaging microscope, using a 100× objective (Carl Zeiss, Jena, Germany). The pictures were treated with the ImageJ software.

## 3. Results

### 3.1. Identification of New Components of the Chlamydial Divisome

**Figure 1:**
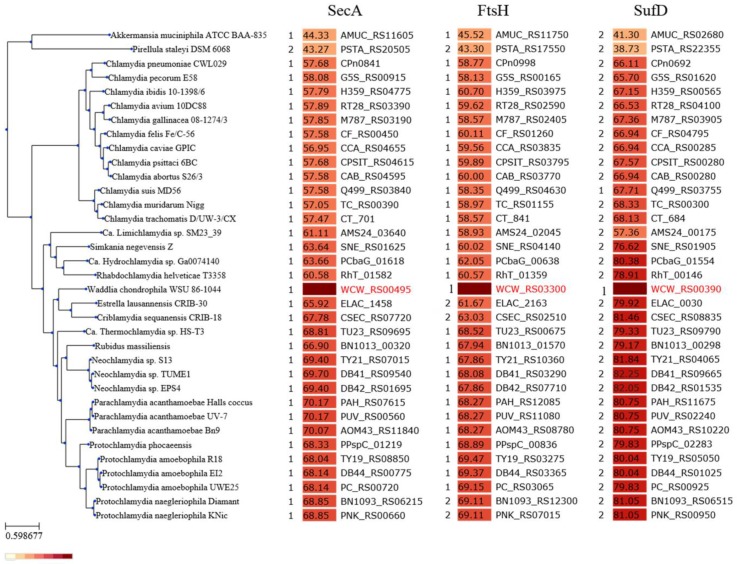
Phylogenic distribution of the proteins of interest among *Chlamydiae.* The phylogenetic tree was reconstructed based on a set of conserved single-copy orthologues, using FastTree 2.1.9. On the left of each column: number of homologs identified in each genome. Color scale from white to red (from low to high): identity of the closest homolog of WCW_0106 (*secA*), WCW_0685 (*ftsH*), and WCW_0087 (*sufD*) that are the focus of this study. The identity was calculated based on multiple sequence alignment made with MAFFT (version 7.058b), as implemented on the chlamdb.ch website [60].

In order to investigate the composition of the chlamydial divisome more in depth, we took advantage of a recent characterization of proteins binding to *W. chondrophila* PG performed in our laboratory [37]. Moreover, we performed a split-ubiquitin Yeast-Two-Hybrid screening [40], with RodZ as a bait (See Material and Methods Section 2.2 for details) (Table S2). We could detect and confirm a large number of potential interactor candidates, among which some are known division proteins or RodZ interactors in other species, such as RodA and FtsK (Table 1). Furthermore, we could select interesting candidates from our previous screening on detection of chlamydial PG-binding proteins in *W. chondrophila* [42]. We then compared the lists of potential interactors and selected promising candidates found in these screens (Table 1). The SecA, FtsH, and SufD proteins (i) that are conserved in all members of the *Chlamydiales* order (for which at least one genome is published) and (ii) that show a potential link with division in literature were selected for further studies. We first confirmed their conservation in all members of the *Chlamydiales* order (Figure 1).

### 3.2. Expression and Localization of RodZ Interactors to the Division Septum 

**Figure 2:**
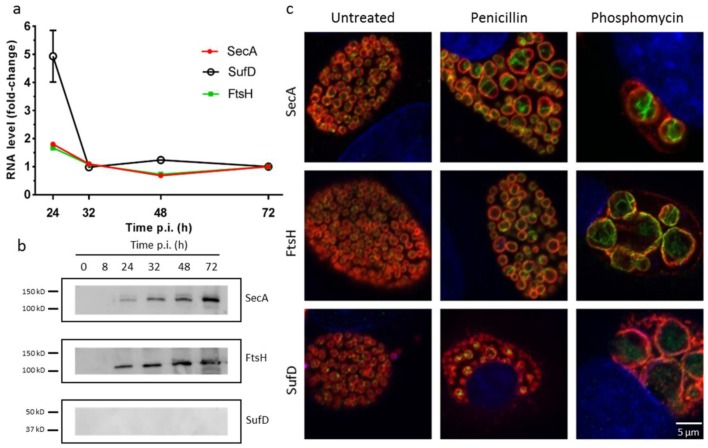
Potential interactors of RodZ are expressed during the developmental cycle. (**a**) RNA expression of *secA*, *sufD*, and *ftsH*, showing more mRNA expression at 24 hours, for all three genes investigated. Vero cells were infected with *W. chondrophila*, and samples were taken at 24, 32, 48, and 72 hours, post-infection. RNA was extracted, cDNA was synthesized, and DNA replication was analyzed by qRT-PCR targeting the 16S rRNA gene. (**b**) Protein expression investigated by Western blotting during the bacterial developmental cycle detected by specific antibodies against SecA, FtsH, and SufD proteins. (**c**) Localization of SecA, FtsH, and SufD in absence of antibiotic treatment and in presence of penicillin or phosphomycin. Vero cells infected with *W. chondrophila* were treated 2 h p.i. (500 µg mL^−1^ phosphomycin or 500 µg mL^−1^ penicillin, and cells were fixed 24 h p.i. Cells were then labeled with a mouse antibody specific to SecA, FtsH, or SufD respectively (green), a rabbit anti *W. chondrophila* antibody (red), and DAPI (blue) and were observed by confocal microscopy.

We then investigated the gene expression pattern of the potential interactors by qRT-PCR. This revealed an increased expression of all three transcripts at 24 h p.i. and lower expression at later time points (Figure 2a). The gene expression of the potential interactors is comparable with the RNA expression pattern of RodZ and MreB [18]. In order to observe the protein expression of the potential division interactors, we raised antibodies against recombinant 6×His-tagged SecA, FtsH, and SufD proteins purified from *E. coli*. Immunoblotting analysis showed that SecA and FtsH are detected as a single band at the predicted size throughout the whole developmental cycle, whereas SufD protein expression was not detected, perhaps due to a poor immunogenicity of this protein (Figure 2b). Next, in order to determine the subcellular localization of the potential RodZ interactors, we performed immunofluorescence on Vero cells infected with *W. chondrophila* (Figure 2c). We could observe a peripheral localization of SecA and FtsH. The peripheral localization is consistent with their putative inner membrane localization and with a possible colocalization with RodZ, which resides in the inner membrane (Figure 2c). We also observed the localization of these proteins in enlarged RBs (ABs) by treating *W. chondrophila* with peptidoglycan synthesis inhibitors, such as penicillin and phosphomycin. Essentially, localization of SecA and FtsH proteins after penicillin treatment is reminiscent of the accumulation of RodZ at aborted division septa in similar conditions [18]. In contrast, the localization of SufD was less clear and would need further investigation (Figure 2c).

### 3.3. Heterologous Overexpression of SecA, FtsH, and SufD Proteins

**Figure 3:**
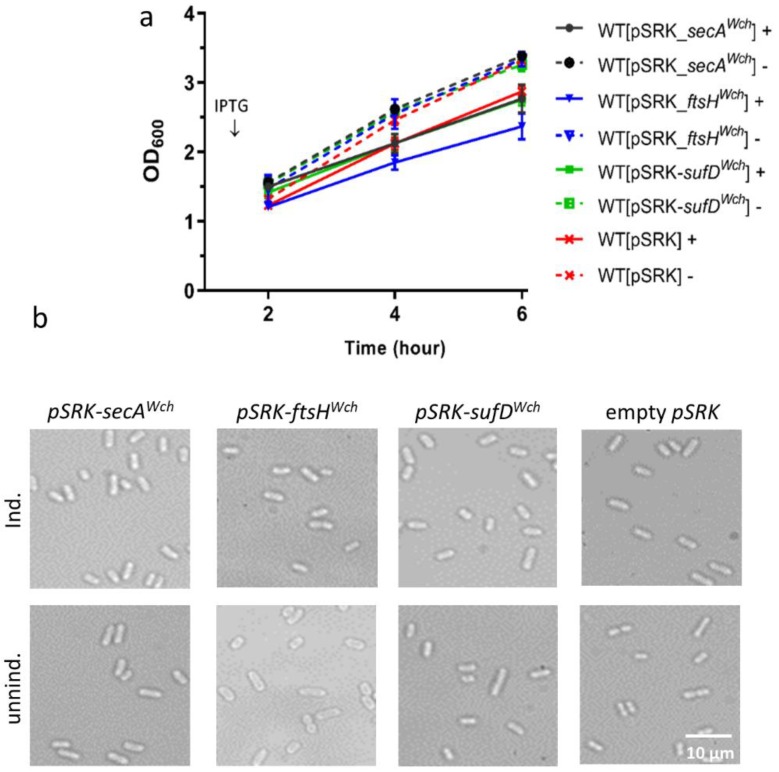
Heterologous overexpression of SecA*^Wch^*, FtsH*^Wch^*, and SufD*^Wch^* in *E. coli.* Solid line (+) with induction, dashed line (-) without induction. (**a**) Growth curve and (**b**) microscopy showing the effect of SecA*^Wch^*, FtsH*^Wch^*, and SufD*^Wch^* overexpression on *E. coli* growth. Overnight cultures were diluted to OD_600_/mL of 0.2. The diluted cultures were incubated for one hour and induced by 1 mM of IPTG; no IPTG was added to the controls. (**a**) Bacterial growth was recorded by measuring the optical density of bacteria every 2, 4, and 6 h, using a spectrophotometer. (**b**) After five hours of induction, the cultures were taken to the microscope and imaged, using a 100× objective.

To get more indications on the involvement of the candidate interactors in bacterial division, we used heterologous overexpression in *E. coli*. A pSRK-Gm plasmid with the *lacP* promoter allowing overexpression of the protein was used [41,61]. The pSRK plasmids containing the genes of interest from *W. chondrophila* were transformed into wild-type *E. coli* and selected using gentamycin. As a control, an empty pSRK plasmid was used (Figure 3). The growth curve pattern of the constructed strains was monitored by turbidity measurements. We observed an impaired bacterial growth and partial inhibition of proliferation during SecA, FtsH, and SufD overexpression, as well as an inhibitory effect of the empty plasmid (Figure 3a). Furthermore, to assess the effect of overexpression on bacterial morphology, the strains were observed by bright-field microscopy. As shown in the Figure 3b, *E. coli* strains overexpressing the proteins of interest did not show any visible morphology defects compared to the uninduced control groups and control strain with the empty plasmid. Thus, overexpression of SecA, FtsH, and SufD from *W. chondrophila* in *E. coli* wild-type does not affect bacterial growth and morphology.

### 3.4. Complementation Studies of the Potential Division Interactors 

To determine the conservation of the gene function between *W. chondrophila* and *E. coli*, we performed plasmid-based complementation studies. The pSRK plasmids coding for the genes of interest from *W. chondrophila* were transformed into their corresponding defective mutants. 

Since *ftsH* and *secA* genes were shown to be essential for numerous bacterial species, construction of null mutants is not trivial. Furthermore, *ftsH^Eco^* has been shown to be essential, and several studies demonstrated the importance of FtsH for *E. coli* growth [62,63]. For the complementation purposes, a construct containing *ftsH^Wch^* was expressed in a *∆ftsH^Eco^* strain [64], growing at 30 °C. *∆ftsH^Eco^* is an *ftsH* null mutant strain with a suppressor mutation in the *sfhC* gene. The mutant strain is expected to have defects in heat-shock response, as well as AAA+ protease function for protein degradation, compared to the wild-type.

Similarly, SecA protein was shown to be essential for the secretion of many vital proteins and crucial for bacterial growth [65,66] and virulence [67]. Thus, we overexpressed *secA^Wch^* in a temperature-sensitive mutant of *E. coli (secA^Eco/ts+^)* [68]. Finally, the knockout (KO) strain of the *sufD^Eco^* was commercially available [69].

First, expression of the proteins of interest in the complemented strains and controls were assessed by immunoblotting (Figure S1). SecA protein of *W. chondrophila* was expressed and showed a band at the right size (SecA-119,387 kDa), but showed neither strong expression nor clear difference in the presence or absence of the inducer. In contrast, FtsH was well expressed in both strains, (FtsH-103,471 kDa), but the expression was stronger upon induction. Similarly, we observed strong SufD expression upon induction (SufD-47,312 kDa).

**Figure 4:**
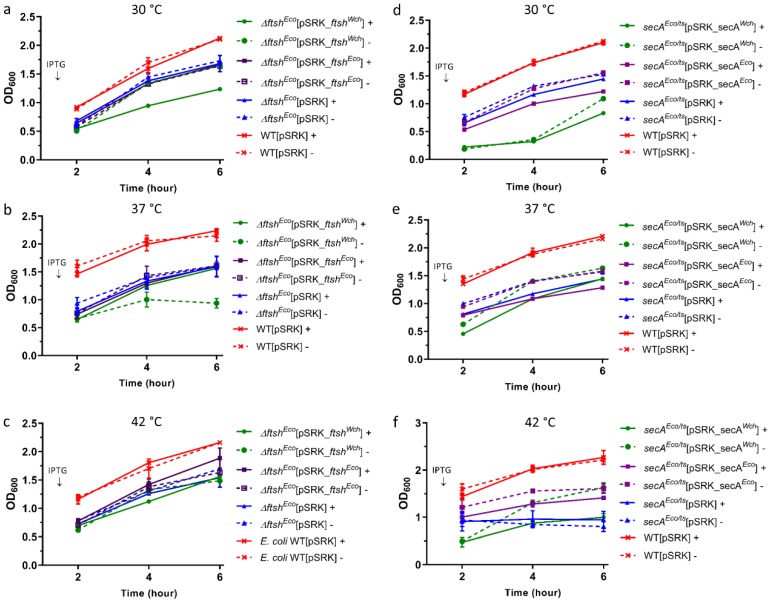
Overexpression of *W. chondrophila* or *E. coli* homologues do not complement growth defects of *E. coli* mutants. Solid line (+) stands for with induction, dashed line (-) stands for without induction. Graphs (**a**–**c**): represent *ftsH^Wch^* and *ftsH^Eco^* overexpression and (**d**–**f**): *secA^Wch^* and *secA^Eco^* overexpression at different temperature conditions: 30, 37, and 42 °C. (**a**,**b**) No restored original phenotype was observed upon *ftsH^Eco^* and *ftsH^Wch^* overexpression in *∆ftsH^Eco^* mutant strain. (c) Slight growth recovery was observed in *∆ftsH^Eco^* mutant expressing at 42 °C *ftsH^Eco^*. (**d**,**e**) No restored original growth phenotype was observed during *secA^Eco^* overexpression in corresponding mutants at 30 °C and 37 °C. (**f**) Slight but not significant growth recovery was observed in *secA^Eco/ts+^* mutants expressing *secA^Eco^* at 42 °C.

Next, we examined the effect of *W. chondrophila* and *E. coli* FtsH, SecA, and SufD expression on bacterial growth and morphology by using corresponding *E. coli* mutants (Figure 4). First, to determine the conditions in which growth defects of the mutant strains are more perceptible, we tested different temperature conditions (Figure 4). The results showed no restored original growth during expression of *ftsH^Wch^* and *ftsH^Eco^* in *∆ftsH^Eco^* (Figure 4a–c). Notably, the expression of *ftsH^Wch^* in *∆ftsH^Eco^* mutant seems to inhibit bacterial growth (Figure 4a,b).

No restored original growth was observed during *secA^Wch^* and *secA^Eco^* expression in *secA^Eco/ts+^* mutant, as well (Figure 4d,e). Furthermore, at 30 °C, *secA^Wch^* expression in the *secA^Eco/ts+^* mutant seems to inhibit bacterial growth (Figure 4d). Finally, *secA^Eco^* expression at 42 °C was the most prominent phenotype (Figure 4f). Thus, upon *secA^Eco^* expression, we could observe a slight growth recovery phenotype, compared to the control expressing empty plasmid.

Results from *sufD^Eco^* and SufD*^Wch^* overexpression in *∆sufD^Eco^* did not show growth phenotype recovery (Figure S2). Since we did not observe a strong phenotype of the *∆sufD^Eco^*, it was also not possible to assess any difference in bacterial growth upon SufD*^Wch^* expression. SufD of *E. coli* was shown to be involved in Fe-S cluster biosynthesis and to play a role in iron acquisition [56]. It was also shown that deletion of SufD abolishes Suf function in vivo [55,70]. Thus, in order to test iron acquisition in the constructs we used the iron chelator 2,2’-Bipyridyl (Figure S3). As we can see, no growth difference was observed between *∆SufD^Eco^* strain alone and upon SufD*^Wch^* overexpression (Figure S3a–c).

**Figure 5:**
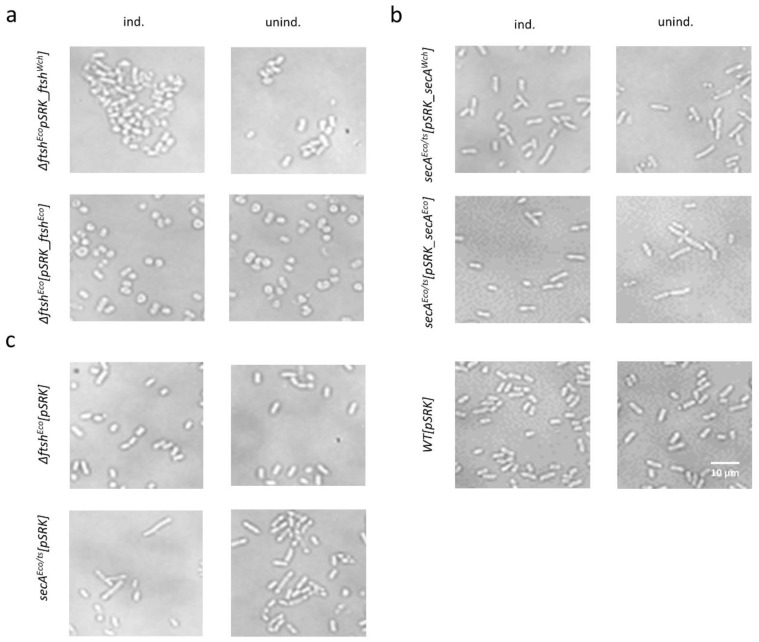
Phenotype of strains overexpressing proteins of interest. After 5 h of incubation at 42 °C, with and without IPTG, the strains were observed by bright-field microscopy. (**a**) *∆ftsH^Eco^* expressing *ftsH^Eco^* and *ftsH^Wch^* caused morphological changes in bacteria shape in presence and absence of IPTG. Bacteria became round and heterogeneously shaped. (**b**) Overexpression of *secA^Eco^* in *secA^Eco/ts+^* temperature-sensitive mutant showed a partial effect on bacterial rod-shape recovery, compared to the slightly elongated control group *secA^Eco/ts+^* not expressing *secA^Eco^*. (**c**) Mutant strains and *E. coli* WT control. Scale bar = 10 µm.

Next, to assess the effect of *Waddlia* and *E. coli* proteins overexpression on bacterial morphology, the strains were observed by bright field microscopy after 6 h of incubation at 42 °C, in the presence and absence of IPTG (Figure 5). We observed minor changes in *∆ftsH^Eco^* mutants complemented with *ftsH^Eco^* and *ftsH^Wch^* in the presence and absence of IPTG (Figure 5). Overexpression of *W. chondrophila* protein in *∆ftsH^Eco^* mutant seems to cause bacterial aggregation (Figure 5a). Different effects were observed in the case of *ftsH^Eco^*: bacteria became round and heterogeneously shaped following the expression of *ftsH^Eco^* in *∆ftsH^Eco^* (Figure 5a).

Taken together, we see no complementation upon expression of FtsH from *W. chondrophila* or *E. coli* in *∆ftsH^Eco^* mutant, but some bacterial aggregation upon overexpression of the *Waddlia* FtsH protein. Expression of *secA^Wch^* and *secA^Eco^* in *secA^Eco/ts+^* mutant seems to have a partial effect on bacterial rod-shape. The rod-shape of bacteria seems to be partially restored upon SecA *^Eco^* overexpression (Figure 5b).

## 4. Discussion

The exact composition of the chlamydial division machinery is not known despite recent advances in the field [15,16,18,26,27,28,29,30,35,36,39,42]. By using the Yeast-Two-Hybrid assay and detecting chlamydial PG-binding proteins with *W. chondrophila* PGLS (peptidoglycan-like structure), potential interactors of the division septum protein RodZ were identified. We show that genes encoding RodZ interactors, SecA, FtsH, and SufD are (i) conserved among all members of the *Chlamydiales* order and are (ii) expressed early during the *W. chondrophila* developmental cycle, which is consistent with the role of these genes in chlamydial division. Moreover, the peripheral localization of SecA and FtsH is consistent with their potential co-localization with RodZ, which is known to localize in the inner membrane. Interestingly, the scenario of RodZ accumulation at aborted division septa in enlarged RBs (ABs) is also repeating in the case of SecA and FtsH interactors. We can speculate that the interactor proteins are recruited to the division site, together with RodZ, to assist in division organization. The exact role of the interactors is not yet known.

SecA protein is known as a member of the universal protein translocation machinery and is conserved in all bacteria. Protein translocation function of SecA might be crucial in transport of the proteins essential for division and division organization. In addition, the SecA protein has already been shown to be localized at the equatorial ring in growing streptococcal chains, a zone of active peptidoglycan synthesis [71]. This is of particular interest since many proteins, such as peptidoglycan modifying enzymes and peptidoglycan-binding proteins, need to be translocated to the periplasm during division. Moreover, it was shown that SecA is presumably required for membrane insertion of RodZ in *E. coli* [72]. It was also suggested that SecA mainly targets the native RodZ to SecYEG, independent of SecB [72]. On the other hand, it was recently shown in *E. coli* that MreB and SecA proteins interact genetically [73]. Upon this interaction, SecA was shown to be a morphogenetic modulator responsible for MreB localization [73]. We can hypothesize that, as a member of the universal protein translocation machinery, SecA might be recruited first at the division septum for RodZ translocation and localization, followed by MreB direction to the midcell and localization as a late recruit [18].

Similarly, FtsH was also shown to be essential in bacteria as an AAA+ protease, which maintains an ATP-driven unfolding and degradation activity of misfolded proteins [74]. Initially, *ftsH* mutant was described as a new temperature-sensitive cell-division mutant causing impaired septation [52]. Essentially, FtsH was shown to accumulate at the midcell in dividing *Bacillus subtilis* and at positions near the cell poles that are the future division sites in sporulating bacteria [53]. FtsH might thus play a role in activation and/or degradation of septal proteins during chlamydial division. All these observations might indicate a potential function of FtsH in bacterial division. Since SpoIID of *W. chondrophila* (a homologue of the protein involved in sporulation in *B. subtilis*) was recently implicated in the division of *Chlamydiales* [39], we hypothesized that FtsH might interact with SpoIID and be implicated in peptidoglycan remodeling, after having initially been recruited at midcell by RodZ.

On its side, SufD is not clearly involved in bacterial division. However, we selected SufD as an interesting candidate because it might bring a link between division regulation and iron deprivation. It was shown that SufD is involved in iron acquisition in *E. coli* [56]. As obligate intracellular pathogens, *Chlamydiales* are dependent on host iron. It was demonstrated that iron deprivation can induce formation of ABs in *C. trachomatis* [57]; therefore, an iron acquisition system is crucial for chlamydial division and survival [57]. We could hypothesize that iron homeostasis in *Chlamydiales* is executed by the SufABCD complex, where SufD is playing an essential role in iron acquisition. This could explain the potential interaction of SufD with RodZ in *W. chondrophila*. Through interacting with RodZ, SufD might have a role in regulating bacterial division. SufD might thus interact with RodZ to inhibit division when iron is not available.

In order to get more evidence of the role of SecA, FtsH, and SufD in bacterial division, we performed heterologous overexpression of the *W. chondrophila* homologues of the potential RodZ interactors in *E. coli*. We hoped that overexpressing these proteins could interfere with the division mechanism of *E. coli*. However, we did not observe any effect of overexpression on bacterial growth and morphology. This lack of phenotype might be explained by strong divergence between chlamydial division and division in *E. coli*. We also did not observe SufD expression in *W. chondrophila* during the course of infection, and this might be due to the low protein level or its instability. These could be investigated further by using a fractionation or extraction protocols to enrich membrane proteins, and this would help localizing native protein.

We performed complementation experiments to better understand whether *W. chondrophila* proteins can fulfill the activity of *E. coli* homologues. We could not test complementation of SufD since we could not find any condition in which the *E. coli ∆sufD* strain had a growth defect (Figure S2). In our system, we could not obtain complementation of *secA^ts^* and *∆ftsH* mutants, neither with the chlamydial homologues nor with the *E. coli* proteins themselves. This might indicate that tight regulation of SecA and FtsH is required for their proper function.

## 5. Conclusions

In conclusion, this study indicates that proteins such as FtsH, SecA, and SufD could interact with chlamydial divisome components and might play a (direct or indirect) role in chlamydial division regulation. Moreover, gene expression profile and subcellular protein localization of the potential interactors are comparable with the aforementioned recently described division septum proteins. We now need further efforts to decipher the role of these proteins and of other potential divisome components in the organization and regulation of the chlamydial divisome.

## Figures and Tables

**Table 1:** Selection of potentially interesting candidates from Yeast-Two-Hybrid hits of RodZ interactors conserved in all members of the *Chlamydiales* order.

Locus_tag	Protein Name	Predicted Function and References
wcw_0302	RodA	Rod-shape determining protein A [43]. Septum-peptidoglycan biosynthetic protein [44]. Member of the SEDS family [45].
wcw_0755	RodZ	MreB membrane anchor [46]. Septal division protein in *Chlamydiales* [18].
wcw_0783	YbbP	Also known as CdaA, major contributor to c-di-AMP synthesis [47]. CdaA of *C. trachomatis* was shown to synthesize c-di-AMP [48].
wcw_1433	FtsK	DNA translocase FtsK [49]. Involved in chromosome segregation during division [50]. During *E. coli* division FtsK interacts with FtsZ [51].
wcw_0685	FtsH	AAA+ protease that degrades misfolded proteins and is involved in cell division [52]. Accumulates at the division septum of *B. subtilis* [53]. Deletion of *ftsH* causes filamentous growth [54].
wcw_0087	SufD	Belongs to the SufBCD complex, responsible for Fe-S cluster biogenesis [55]. Deletion of SufD abolishes Suf function in vivo and reduces bacteria survival [56]. Iron deprivation in *C. trachomatis* blocks division and induces formation of aberrant bodies [57].
wcw_0357	YaeL	Protease, essential for cell growth [58]. Depletion causes filamentation [59].

## Supplementary Materials

The following are available online at https://www.mdpi.com/2076-2607/7/12/617/s1. Figure S1: Immunoblotting analyses of protein expression in the complemented strains. Figure S2: Bacterial growth curve pattern expressing *Waddlia* homologues of interest. Figure S3: Treatment with an iron chelator, 2, 2’-Bipyridyl. Table S1: Primers used in this study. Table S2a,b: Yeast-Two-Hybrid hits of RodZ interactors.
