# Supplementary material for: Interactions Screenings Unearth Potential New Divisome Components in the Chlamydia-Related Bacterium, Waddlia chondrophila
Source: Microorganisms. 2019 Nov 26;7(12):617. doi: 10.3390/microorganisms7120617 (PMC6956297; doi:10.3390/microorganisms7120617)
Supplement: Supplementary file 1 [file microorganisms-07-00617-s001.pdf]

Supplementary Material

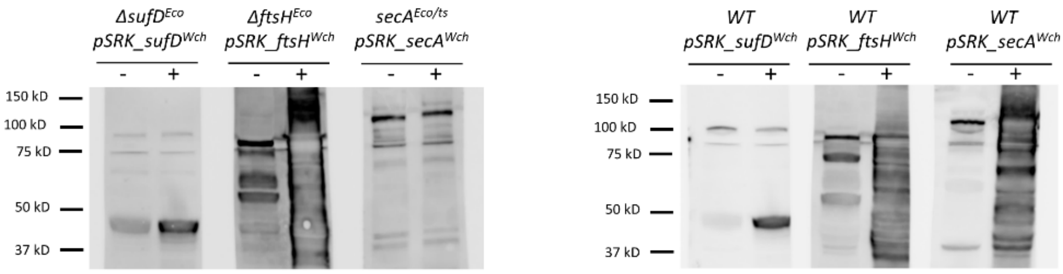

Figure 1. Immunoblotting analyses of protein expression in the complemented strains (+) with and (−) without induction, using antibodies raised against recombinant 6xHis-tagged SecA, FtsH, and SufD. SecA, SufD and FtsH proteins of *W. chondrophila* were detected at the right size: SecA-119,387 kDa, FtsH-103,471 kDa, SufD-47,312 kDa.

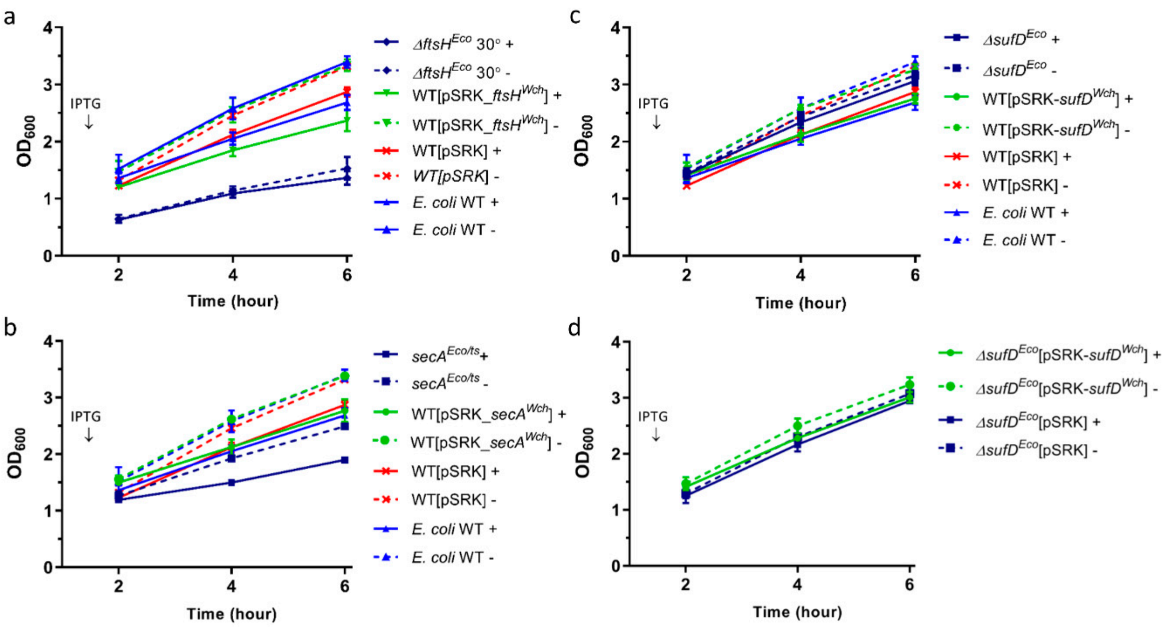

Figure 2. Bacterial growth curve pattern expressing *Waddlia* homologues of interest. Solid line (+) with induction, dashed line (−) without induction. (a, b) *secA*^{Wch} and *ftsH*^{Wch} overexpression in corresponding mutants shows no restored original growth phenotype. (c, d) No restored original phenotype was observed upon *sufD*^{Wch} overexpression.

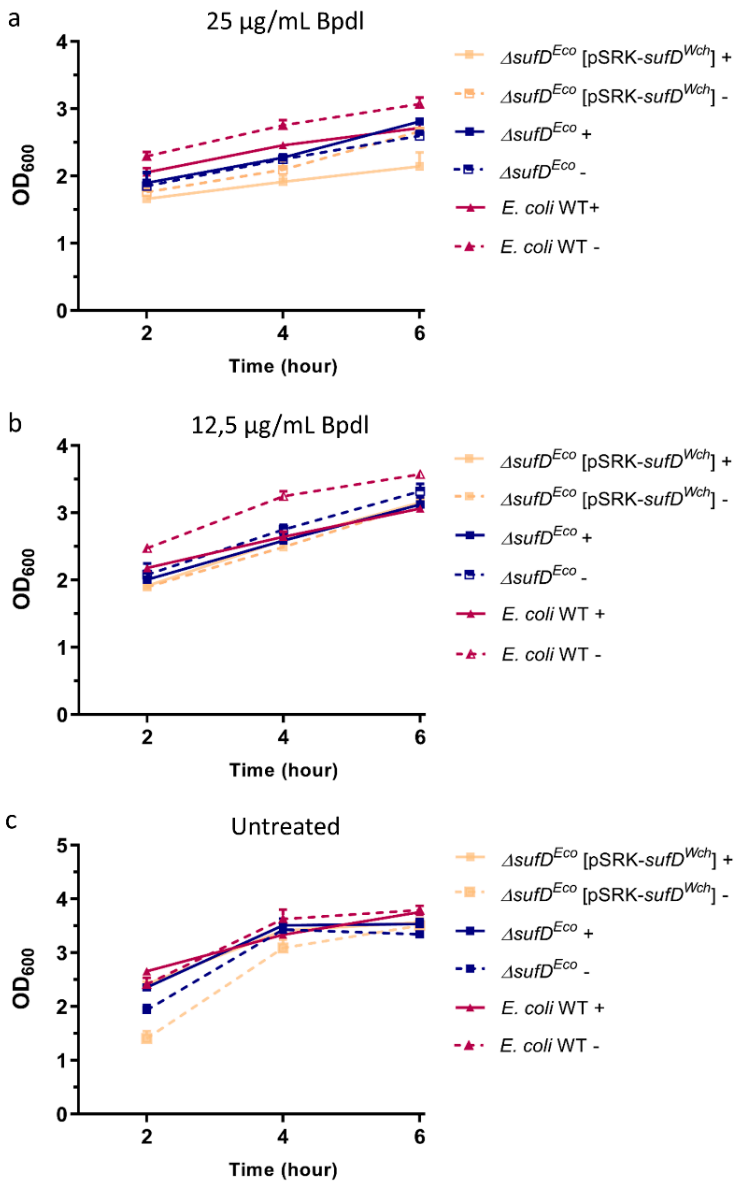

Figure 3. Treatment with an iron chelator, 2, 2′-Bipyridyl. Solid line (+) with induction, dashed line (−) without induction. The overnight cultures grown at 37 °C were diluted to an absorbance at 600nm (OD_{600}) of 0.05. The diluted cultures were incubated for 1 hour to exponential phase and induced by 1 mM of IPTG (no IPTG was added to the controls) and incubated 1 more hour after the addition of 2, 2′-Bipyridyl addition. Two concentrations of 2, 2′-Bipyridyl were used, (a) 25 µg/mL, (b) 12, 5 µg/mL and (c) control group without an iron chelator. No difference in bacterial growth was observed upon *SufD*^{Wch} overexpression, independent on the iron chelator concentration.

Table 1. Primers used in this study.

FtsH_W_ch_F	5`-TTTGGCAAAGAACGACGCAG-3`
FtsH_W_ch_R	5`-TCGGAATGTTCGACAGCCAA-3`
SecA_W_ch_F	5`-TTGCTGGACAATTTGCTGCC-3`
SecA_W_ch_R	5`-TCAGCGTTTTTCCCTCTCCC-3`
SufD_W_ch_F	5`-ACCGATCCTTTTGCCGTTCT-3`
SufD_W_ch_R	5`-TGCAGACGAGGCTGTACAAG-3`
ftsH_wch_NdeI_F	5`-AAAAAACATATGGCTGACGATAAACAAGATTTT-3`
ftsH_wch_XbaI_R	5`-AAAAAATCTAGATTAAGCTCCTAGATCGCTTCT-3`
secA_wch_NdeI_F	5`-AAAAAACATATGATCAGTTTTTTCAAGAAATT-3`
secA_wch_XbaI_R	5`-AAAAAATCTAGATTATTCGTCAGCCTGATGG-3`
sufD_wch_NdeI_F	5`-AAAAAACATATGACTCTTTTCGAGGAACAATTGA-3`
sufD_wch_NotI_R	5`-AAAAAAGCGGCCGCTTACCAACCTTTTGTTATTTCATCCCT-3`
Ftsh_K-12_SacI_F	5`-AAAAAAGAGCTCATGGCGAAAAACCTAATACTCTGG-3`
Ftsh_K-12_XbaI_R	5`-AAAAAATCTAGATTACTTGTCGCCTAACTGCTCTG-3`
SecA_K-12_NdeI_F	5`-AAAAAACATATGCTAATCAAATTGTTAACTAAAGTT-3`
SecA_K-12_XbaI_R	5`-AAAAAATCTAGATTATTGCAGGCGGCCATG-3`
sufD_K-12_NdeI_F	5`-AAAAAACATATGGCTGGCTTACCGAACAG-3`
sufD_K-12_XbaI_R	5`-AAAAAATCTAGATCATCTTGCACCTCCTGGCA-3`

Table 2. Yeast Two Hybrid hits of RodZ interactors. Split-ubiquitin Yeast-Two-Hybrid screening has been performed with RodZ as a bait and proteins interacting with RodZ have been identified. The domain annotation was retrieved from the online database chlamdb.ch [43].

Table S2. (a). Yeast Two Hybrid hits of RodZ interactors. A total of 28 putative RodZ interactors conserved in all members of the *Chlamydiales* order.

Locus_tag	Protein name	Occurrence in the screen	Product description	Predicted domains	Similarity
wcw_0302	RodA	4	rod-shape determining protein A. Septum-peptidoglycan biosynthetic protein	PFPF01098: cell cycle protein	belongs to the SEDS family
wcw_0755	RodZ	4		PF13413: helix-turn-helix domain	
wcw_0783	YbbP	4	conserved hypothetical membrane protein	PFPF02457: DisA bacterial checkpoint controller nucleotide-binding	belongs to the adenylate cyclase family. DacA/CdaA subfamily
wcw_1433	FtsK	3	DNA translocase ftsK	PFPF17854: FtsK alpha domain<br>PFPF01580: FtsK/SpoIIIE family<br>PFPF09397: Ftsk gamma domain	belongs to the FtsK/SpoIIIE/SftA family
wcw_0216	NqrF	2	Na(+)-translocating NADH-quinone reductase subunit F	PFPF00970: oxidoreductase FAD-binding domain<br>PFPF00111: 2Fe-2S iron-sulfur cluster binding domain	belongs to the NqrF family
wcw_0685	FtsH	2	putative cell division protein FtsH	PFPF01434: peptidase family M41<br>PFPF17862: AAA+ lid domain<br>PFPF00004: ATPase family associated with various cellular activities (AAA)	belongs to the AAA ATPase family
wcw_1947	-	2	conserved hypothetical protein	PFPF18095: UPF0242 C-terminal PAS-like domain<br>PFPF06785: uncharacterised protein family (UPF0242) N-terminus	-
wcw_0087	SufD	1	FeS assembly protein	PFPF01458: uncharacterized protein family (UPF0051)	-
wcw_0231	Amn	1	AMP nucleosidase	PFPF01048: phosphorylase superfamily	-
wcw_0270	NqrB	1	Na(+) -translocating NADH-quinone reductase subunit B	PFPF03116: NQR2, RnfD, RnfE family	belongs to the NqrB/RnfD family
wcw_0357	YaeL	1	metalloprotease	PFPF02163: peptidase family M50	-
wcw_0674	-	1	hypothetical protein	PFPF00589: phage integrase family	-
wcw_0681	-	1	putative permease, YjgP/YjgQ family	PFPF03739: lipopolysaccharide export system permease LptF/LptG	-
wcw_0738	GatA	1	glutamyl-tRNA(Gln) amidotransferase subunit A	PFPF01425: amidase	belongs to the amidase family. GatA subfamily
wcw_0896	-	1	putative membrane protein	PFPF00892: EamA-like transporter family	-
wcw_0998	SucB	1	2-oxogluturate dehydrogenase complex E2 component	PFPF00364: biotin-requiring enzyme<br>PFPF00198: 2-oxoacid dehydrogenases acyltransferase (catalytic domain)	-
wcw_1012	GyrA	1	DNA gyrase subunit A	PFPF03989: DNA gyrase C-terminal domain, beta-propeller	belongs to the topoisomerase GyrA/ParC subunit family
wcw_1302	AroA	1	3-phosphoshikimate 1-carboxyvinyltransferase	PFPF00275: EPSP synthase (3-phosphoshikimate 1-carboxyvinyltransferase)	belongs to the EPSP synthase family
wcw_1334	TyrS	1	tyrosyl-tRNA synthetase	-	belongs to the class-I aminoacyl-tRNA synthetase family. TyrS type 1 subfamily
wcw_1428	-	1	conserved hypothetical protein	PFPF09346:§ SMI1/KNR4 family (SUKH-1)	-
wcw_1442	Lon1	1	Lon ATP-dependent proteaseATP-dependent protease	PFPF00004: ATPase family associated with various cellular activities (AAA)<br>PFPF02190: ATP-dependent protease La (LON) substrate-binding domain<br>PFPF05362: Lon protease (S16) C-terminal proteolytic domain	belongs to the peptidase S16 family
wcw_1535	-	1	conserved hypothetical protein	-	-
wcw_1564	HemA	1	putative glutamyl-tRNA reductase	PFPF01488: shikimate/quinate 5-dehydrogenase<br>PFPF05201: glutamyl-tRNAGlu reductase, N-terminal domain	belongs to the glutamyl-tRNA reductase family
wcw_1647	Rho	1	transcription termination factor rho	PFPF07497: Rho termination factor, RNA-binding domain<br>PFPF00006: ATP synthase alpha/beta family, nucleotide-binding domain<br>PFPF07498: Rho termination factor, N-terminal domain	belongs to the Rho family
wcw_1648	-	1	conserved hypothetical protein	-	-
wcw_1670	-	1	ABC-type transporter, permease subunit	PFPF00950: ABC 3 transport family	belongs to the ABC-3 integral membrane protein family
wcw_1849	GroEL3	1	chaperonin GroEL	PFPF00118: TCP-1/cpn60 chaperonin family	belongs to the chaperonin (HSP60) family
wcw_1869	PepP	1	aminopeptidase P	PFPF01321: creatinase/Prolidase N-terminal domain<br>PF: PF00557 Metallopeptidase family M24	-

Table 2. (b). Yeast Two Hybrid hits of RodZ interactors. A total of 44 putative RodZ interactors not conserved in all members of the *Chlamydiales* order.

Locus_tag	Protein name	Occurrence in the screen	Product description	Predicted domains	Similarity
wcw_1322	MrpC	62	multiple resistance and pH homeostasis system	PFPF00420: NADH-ubiquinone/plastoquinone oxidoreductase chain 4L	-
wcw_0480	NorB	15	nitric oxid reductase	PFPF00115: cytochrome C and quinol oxidase polypeptide I	-
wcw_0273	-	10	conserved hypothetical protein	PFPF03729: short repeat of unknown function (DUF308)	-
wcw_1213	-	10	putative membrane-bound serine protease	PFPF01972: serine dehydrogenase proteinase	-
wcw_1726	-	9	putative transporter	PFPF02080: TrkA-C domain<br>PFPF03600: citrate transporter	-
wcw_0015	OpgH	7	putative glucan biosynthesis	PFPF13632: glycosyl transferase family group 2	-
wcw_0041	-	6	hypothetical protein	-	-
wcw_1781	-	6	transferase	PFPF13231: dolichyl-phosphate-mannose-protein mannosyltransferase	-
wcw_1765	-	4	putative transporter membrane protein	PFPF04632: fusaric acid resistance protein family	-
wcw_0987	-	3	putative mechanosensitive channel	PFPF00924: mechanosensitive ion channel	-
wcw_1725	-	3	Ca^{2+}/Na^{+} antiporter	PFPF01699: sodium/calcium exchanger protein	-
wcw_1818	Wzx	3	O-antigen translocase	PFPF13440: polysaccharide biosynthesis protein	-
wcw_0648	-	2	hypothetical protein	-	-
wcw_1052	-	2	hypothetical protein	-	-
wcw_1101	HtpX	2	putative heat shock protein HtpX	PFPF01435: peptidase family M48	-
wcw_1136	-	2	putative sensory histidine kinase	PFPF13426: PAS domain<br>PFPF02518: histidine kinase-, DNA gyrase B-, and HSP90-like ATPase<br>PFPF03924: CHASE domain<br>PFPF00512: His Kinase A (phospho-acceptor) domain<br>PFPF08448: PAS fold	-
wcw_1311	-	2	putative membrane protein	-	-
wcw_1321	MrpD	2	multiple resistance and pH homeostasis system	PFPF00361: proton-conducting membrane transporter	-
wcw_1577	-	2	hypothetical protein	-	-
wcw_1717	YbhS	2	inner membrane transport permease YbhS	PFPF12698: ABC-2 family transporter protein	-
wcw_p0003	-	1	hypotetical protein	-	-
wcw_0139	-	1	putative trehalose/maltose hydrolase	PFPF03636: glycosyl hydrolase family 65, N-terminal domain<br>PFPF03633: glycosyl hydrolase family 65, C-terminal domain<br>PFPF03632: glycosyl hydrolase family 65 central catalytic domain	-
wcw_0165	-	1	hypothetical protein	-	-
wcw_0326	-	1	putative membrane protein	PFPF13519: von Willebrand factor type A domain	-
wcw_0341	PyrB	1	aspartate carbamoyltransferase, catalytic subunit	PFPF00185: aspartate/ornithine carbamoyltransferase, Asp/Orn binding domain<br>PFPF02729: aspartate/ornithine carbamoyltransferase, carbamoyl-P binding domain	belongs to the ATCase/OTCase family
wcw_0399	-	1	hypothetical protein	-	-
wcw_0409	-	1	hypothetical protein	PFPF13166: AAA domain	-
wcw_0485	CyoE	1	protoheme IX farnesyltransferase	PFPF01040: UbiA prenyltransferase family	belongs to the UbiA prenyltransferase family. Protoheme IX farnesyltransferase subfamily
wcw_0495	-	1	putative bumetanide-sensitive Na-K-Cl cotransporter	PFPF03522: solute carrier family 12<br>PFPF00324: amino acid permease	-
wcw_0514	AdhC	1	NADP-dependent alcohol dehydrogenase C	PFPF00107: Zinc-binding dehydrogenase<br>PFPF08240: alcohol dehydrogenase GroES-like domain	belongs to the zinc-containing alcohol dehydrogenase family
wcw_0692	-	1	putative membrane protein	-	-
wcw_0756	-	1	conserved putative membrane protein	PFPF04018: domain of unknown function (DUF368)	-
wcw_0799	-	1	permease	PFPF00860: permease family	-
wcw_1233	RadC	1	DNA repair protein radC family	PFPF04002: RadC-like JAB domain	belongs to the UPF0758 family
wcw_1528	-	1	conserved hypothetical protein	-	-
wcw_1569	-	1	hypothetical protein	-	-
wcw_1620	SctQ3	1	putative type III secretion translocase SctQ	PFPF01052: type III flagellar switch regulator (C-ring) FliN C-term	-
wcw_1631	-	1	hypothetical protein	-	-
wcw_1699	Ntt5	1	putative ADP/ATP translocase	PFPF03219: TLC ATP/ADP transporter	belongs to the ADP/ATP translocase tlc family
wcw_1827	AcrB	1	multidrug efflux transport protein	PFPF00873: AcrB/AcrD/AcrF family	belongs to the resistance-nodulation-cell division (RND) (TC 2.A.6) family
wcw_1844	OmpA11	1	putative membrane protein	PFPF05150: *Legionella pneumophila* major outer membrane protein precursor	-
wcw_1906	-	1	putative membrane protein	-	-
wcw_1919	YnaI	1	putative mscS family protein	PFPF00924: mechanosensitive ion channel	-
wcw_1968	-	1	conserved hypothetical protein	PFPF00355: Rieske [2Fe-2S] domain	-
